# Combined Whole-Cell High-Throughput Functional Screening for Identification of New Nicotinamidases/Pyrazinamidases in Metagenomic/Polygenomic Libraries

**DOI:** 10.3389/fmicb.2016.01915

**Published:** 2016-11-29

**Authors:** Rubén Zapata-Pérez, Antonio G. García-Saura, Mohamed Jebbar, Peter N. Golyshin, Álvaro Sánchez-Ferrer

**Affiliations:** ^1^Department of Biochemistry and Molecular Biology-A, Faculty of Biology, Regional Campus of International Excellence “Campus Mare Nostrum”, University of MurciaMurcia, Spain; ^2^Univ Brest, CNRS, Ifremer, UMR 6197-Laboratoire de Microbiologie des Environnements Extrêmes (LM2E), Institut Universitaire Européen de la Mer (IUEM)Plouzané, France; ^3^School of Biological Sciences, Bangor UniversityBangor, UK; ^4^Immanuel Kant Baltic Federal UniversityKaliningrad, Russia; ^5^Murcia Biomedical Research InstituteMurcia, Spain

**Keywords:** amidohydrolases, nicotinamidase, functional screening, metagenome, library, high-throughput screening assays

## Abstract

Nicotinamidases catalyze the hydrolysis of the amide bond in nicotinamide (NAM) to produce ammonia and nicotinic acid (NA). These enzymes are an essential component of the NAD^+^ salvage pathway and are implicated in the viability of several pathogenic organisms. Its absence in humans makes them a promising drug target. In addition, although they are key analytical biocatalysts for screening modulators in relevant biomedical enzymes, such as sirtuins and poly-ADP-ribosyltransferases, no commercial sources are available. Surprisingly, the finding of an affordable source of nicotinamidase from metagenomic libraries is hindered by the absence of a suitable and fast screening method. In this manuscript, we describe the development of two new whole-cell methods using the chemical property of one of the products formed in the enzymatic reaction (pyrazinoic or NA) to form colored complexes with stable iron salts, such as ammonium ferrous sulfate or sodium nitroprusside (SNP). After optimization of the assay conditions, a fosmid polygenomic expression library obtained from deep-sea mesophilic bacteria was screened, discovering several positive clones with the ammonium ferrous sulfate method. Their quantitative rescreening with the SNP method allowed the finding of the first nicotinamidase with balanced catalytic efficiency toward NAM (nicotinamidase activity) and pyrazinamide (pyrazinamidase activity). Its biochemical characterization has also made possible the development of the first high-throughput whole-cell method for prescreening of new nicotinamidase inhibitors by the naked eye, saving time and costs in the design of future antimicrobial and antiparasitic agents.

## Introduction

**FIGURE 1:**
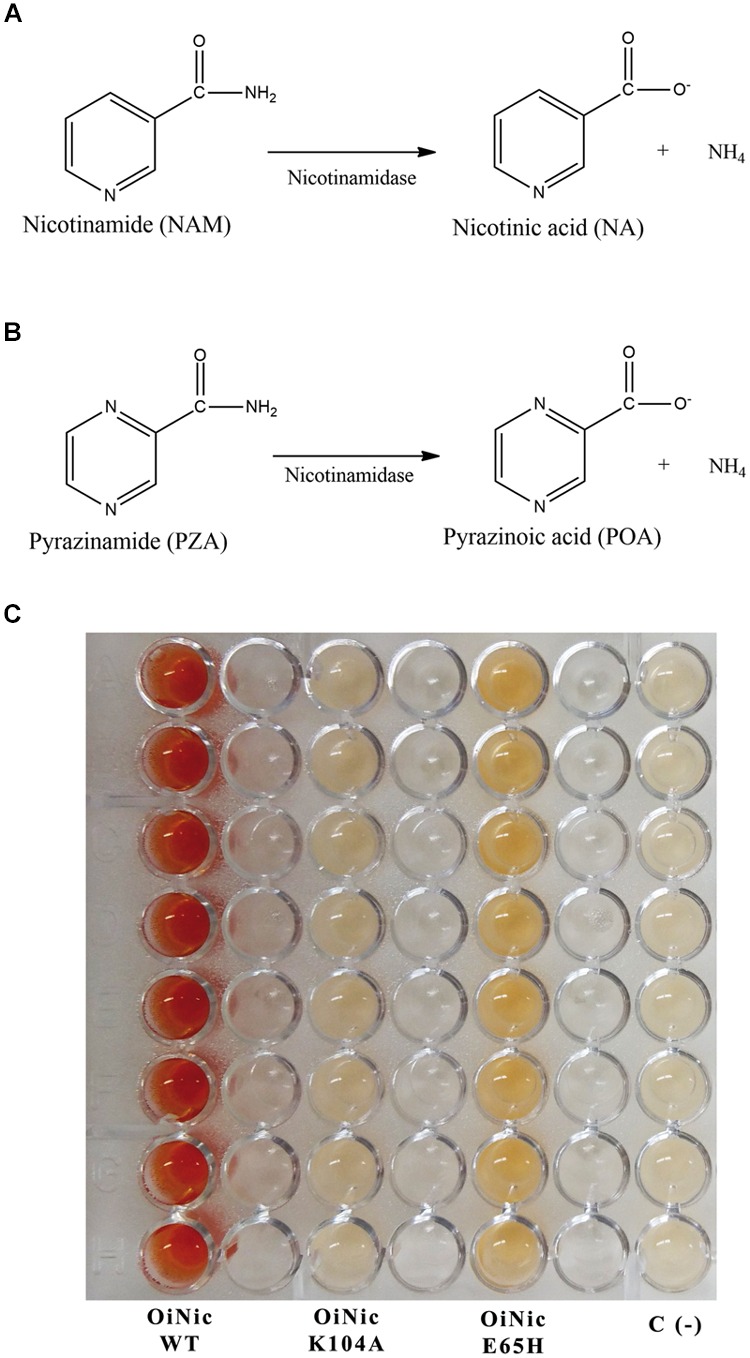
**Reactions catalyzed by nicotinamidases and their use to develop a screening method. (A)** Nicotinamidase activity toward nicotinamide (NAM). **(B)** Pyrazinamidase activity toward pyrazinamide (PZA). **(C)** Reaction plate obtained using the pyrazinamide/ammonium ferrous sulfate (PZA/AFS) method with different recombinant clones. Nicotinamidase clones were *Oceanobacillus iheyensis* nicotinamidase (OiNic Wt) and its corresponding K104A and E65H mutants in pET28a vectors (Sanchez-Carron et al., 2013). Control wells were *E. coli* Rosetta 2 transformed with the same pET empty vector. Color was developed after re-suspending cell pellets in a mixture of 20 mM PZA and 1% AFS for 1 h at 37°C.

Nicotinamidases (EC 3.5.1.19) are key metal-dependent amidohydrolases in NAD^+^ metabolism of multiple species of archaea (Stekhanova et al., 2014), bacteria (Frothingham et al., 1996; Boshoff and Mizrahi, 1998; Zhang et al., 2008; French et al., 2010b; Sanchez-Carron et al., 2013), yeast (Joshi and Handler, 1962; Hu et al., 2007), protozoa (Zerez et al., 1990; Gazanion et al., 2011) and plants (Wang and Pichersky, 2007) that catalyze the hydrolysis of nicotinamide (NAM) (**Figure 1A**) and its analog pyrazinamide (PZA) (**Figure 1B**) to nicotinic acid (NA) or pyrazinoic acid (POA) and ammonia, respectively. They are also present in many metazoans such as *Drosophila melanogaster* (Balan et al., 2008) and *Caenorhabditis elegans* (van der Horst et al., 2007), but absent in mammals, since they alternatively use NAM phosphoribosyltransferase (NAMPT) to convert NAM directly to NAM mononucleotide (NMN), which is then recycled to NAD^+^ (Opitz and Heiland, 2015). This conspicuous absence of nicotinamidases in humans makes them a promising drug target, especially in infections caused by *Borrelia burgdorferi* (involved in Lyme disease) (Purser et al., 2003), *Brucella abortus* (etiological agent of Malta fever) (Kim et al., 2004), *Plasmodium falciparum* protozoa (associated with malarial death) (Zerez et al., 1990) and *Leishmania infantum* protozoa (causative agent of infantile visceral leishmaniasis) (Gazanion et al., 2011), in which this enzyme has been shown to be essential for the survival of these pathogenic organisms (Mesquita et al., 2016).

Furthermore, nicotinamidases have become a strategic focus to be applied as efficient analytical biocatalysts to determine the activity of various classes of relevant biomedical enzymes, including NAD^+^-dependent deacetylases (sirtuins), CD38 and related glycohydrolases, PARPs and mono-ADP-ribosyltransferases, PBEF/NAMPT and similar enzymes, such as NMN adenylyltransferase (NMNAT) and NAM riboside kinase (NRK) (Hubbard and Sinclair, 2013). Those nicotinamidase-coupled methods have also been used to identify modulators of the above classes of enzymes, which are involved in lifespan, cancer, obesity, and neurodegenerative diseases [for reviews of such modulators see (Chen, 2011; Chen et al., 2015; Lord et al., 2015)] as well as to identify levels of NAM, β-NAD, NMN, and NAM riboside (NR) in clinical samples. However, all these clinical and analytical applications have been restricted to an academic environment, since no commercial nicotinamidases are available, and a simple and inexpensive source of enzyme has not been found yet. In this sense, functional metagenomics has arisen as a powerful tool (Mirete et al., 2016), since it allows, on one hand, the discovery of novel biocatalysts of uncultured bacteria, thus circumventing traditional methods that rely on cultivation, and on the other hand, avoids the major drawback of sequence-based screening technologies, which do not allow direct conclusions about the functionality and biochemical parameters of the encoded enzymes. Nevertheless, functional metagenomics has the important limitation of setting up function-driven screening assays (Martínez-Martínez et al., 2015), which in most cases are very tedious and time-consuming, requiring complex substrates and sophisticated chromogenic assays as well as high-performance liquid chromatography (HPLC) or similar analytical methods. Thus, it is not surprising that the majority of metagenome-derived enzymes that have been biochemically characterized are mainly esterases/lipases and glycoside hydrolases (Lopez-Lopez et al., 2014; Sathya and Khan, 2014; Ufarte et al., 2015).

To solve the above-described problems, we report a combination of two high-throughput screening (HTS) assays, which are amenable to identify metagenomic nicotinamidases. The first assay is based on a modification of an already known method (Wayne, 1974), which consists of the incubation of whole cells with PZA and ammonium ferrous sulfate (AFS), and allows a rapid and perfect contrast between positive and negative clones, even with the naked eye. In addition, the second assay is a new whole-cell method based on a different stable iron salt, sodium nitroprusside (SNP). This latter method is able to detect clones not only with high activity toward PZA, but also toward NAM. Using the first method, we have screened a fosmid library with 8000 clones obtained from mesophilic marine bacteria (MB polygenomic library) and thereby identified some positive clones that showed significant pyrazinamidase activity. These positive clones were rescreened with the second method, finding a novel enzyme designated as PolyNic, which was highly active toward PZA, NAM and its analogs, and for the first time ever, with balanced nicotinamidase and pyrazinamidase activities. Finally, after its biochemical characterization, the first method was also optimized for qualitative screening of new possible nicotinamidase inhibitors.

## Materials and Methods

### Strains, Plasmids, and Chemicals

*Escherichia coli* EPI300-T1 (Epicentre, Madison, WI, USA) was used for the screening of the genomic libraries cloned in pCC1FOS fosmids library (Epicentre). *E. coli* strain Rosetta 2 (DE3) was used as the host for pET46 Ek/LIC expression vector. Nicotinamidase substrates, inhibitors, and stable iron salts were obtained from Sigma-Aldrich.

### Genomic DNA Libraries

Fosmid library contained inserts of mixed genomic DNA of 194 mesophilic bacteria (MB) isolated from deep-sea hydrothermal vents sampled at various sites in Mid Atlantic Ridge. The DNA extracted from individual isolates was pooled in equal ratios and used to prepare a polygenomic library using Epicentre Fosmid Library Construction Kit (Epicentre), according to the manufacturer instructions and as described earlier, to produce a library named MB (Leis et al., 2015). The fosmids clones in *E. coli* EPI300-T1 were arrayed in 384-well microtiter plates and stored at -80°C in LB with chloramphenicol (12.5 μg/mL).

### Functional Screening

To detect pyrazinamidase activity in the fosmid constructions of the polygenomic libraries, we used a modification of the Wayne’s pyrazinamidase assay (Wayne, 1974). This method is based on the complex formed between AFS and POA formed in the pyrazinamidase reaction, giving rise to an intense red color. Briefly, *Escherichia coli* EPI300-T1 transformed with the fosmid library was grown on LB agar plates at 37°C overnight to yield single colonies, which were stored in 384-well plates with LB and chloramphenicol (12.5 μg/mL) at -80°C. Replica plating was used to transfer the library colonies to 96-well plates containing 200 μL of fresh LB with the same antibiotic and Fosmid Autoinduction Solution (Epicentre, Madison, WI, USA) for activation of the *oriV* origin and hence for increasing of fosmids copy number per cell. After growing at 37°C overnight, their pellets were then re-suspended in a mixture of 20 mM PZA and 1% AFS dissolved in MilliQ^®^ water, and incubated for 1 h at 37°C until an intense red color appeared. If desired, the revealed plates could be incubated overnight to obtain an even more intense red color with no appreciable change in control wells. Positive and negative controls were made using previously cloned *Oceanobacillus iheyensis* nicotinamidase (OiNic) (Sanchez-Carron et al., 2013) and empty EPI300-T1 cells, respectively.

To detect pyrazinamidase or nicotinamidase activity using SNP, culture conditions were the same as in the previous method (AFS method), but pellets were re-suspended in 1 mM PZA or NAM with 0.75% SNP. Once re-suspended, plates were incubated at 37°C for 4 h, centrifuged, and the supernatants were transferred to flat 96-well plates. The difference in absorbance between the negative control wells and those containing the fosmids clones was measured at 495 or 395 nm, to detect pyrazinamidase or nicotinamidase activity, respectively.

### Sequence Analysis

Positive clones were selected and their DNA inserts were sequenced using a Roche 454 GS FLX Ti sequencer (454 Life Sciences, Branford, CT, USA) at Life Sequencing SL (Valencia, Spain). Upon completion of sequencing, the reads were assembled to generate non-redundant meta-sequences using Newbler GS De Novo Assembler v.2.3 (Roche). The GeneMark software (Lukashin and Borodovsky, 1998) was employed to predict potential protein-coding regions (open reading frames (ORFs) with ≥20 amino acids) from the sequences of each assembled contig, and deduced proteins were screened via blastp and psi-blast (Altschul et al., 1997). ESPript (Robert and Gouet, 2014) was used for displaying alignments. PolyNic homology modeling was carried out with Swiss-Model^1^ (Biasini et al., 2014) using *Pyrococcus horikoshii* nicotinamidase coordinates as template (PhNic, PDB 1IM5), and structurally aligned with Chimera (Pettersen et al., 2004).

### Cloning, Expression, and Purification

The selected positive gene of the fosmid library (contig 00048_2428, NCBI’s BioSample accession: SAMN05386332 and NCBI’s Sequence Read Archive accession: SRP079696), named as *PolyNic*, was amplified by PCR with a high fidelity thermostable DNA polymerase (Pfu UltraII Fusion HS, Agilent). The respective forward and reverse oligonucleotides were 5′-ATGGAAATAAAGGAGAAATTCACCGTGTCGCCAACTCTCCATTATCAACCTAATGACGCG-3′ and 5′-CTACTGGTCGTGGACATGCAACGCGACTTCTGCGAAGGCGGAGCTCTGGCGATTGATGGC-3′. The amplified PCR product (588 bp) was inserted into pET-46 Ek/LIC vector (EMD Millipore). The vector was later transformed into *E. coli* Rosetta 2 (DE3). Cells were grown in 1 L of Terrific Broth (TB) supplemented with ampicillin (50 μg/mL) and chloramphenicol (12.5 μg/mL) and, when the OD_600_ reached a value of 4, induced with 0.5 mM isopropyl-β-thiogalactoside (IPTG) for 16 h at 25°C with constant agitation. Cells were harvested by centrifugation, re-suspended in lysis buffer (50 mM sodium phosphate buffer pH 7.3 containing 300 mM NaCl) and disrupted using a Bead Beater (BioSpec, USA). The protein in the supernatant was then purified by Ni^2+^-chelating affinity chromatography (ÄKTA Prime Plus, GE Life Sciences) onto a HiPrep IMAC 16/10 FF 20 mL column (GE Life Sciences). Fractions containing the desired protein were desalted, concentrated and filtrated onto a Superdex 200 HiLoad 16/600 column (GE Life Sciences), obtaining an electrophoretically pure enzyme. The protein molecular mass was determined by SDS-PAGE, gel filtration and using an HPLC/ESI/ion trap system (Sanchez-Carron et al., 2011). Protein concentration was determined using Bradford reagent (Bio-Rad) and BSA as standard.

### Enzyme Assay

Nicotinamidase activity was determined chromatographically by HPLC following the increase in the NA area, using an HPLC (Agilent 1100 series) with a reverse-phase C_18_ 250 mm × 4.6 mm column (Gemini C18, Phenomenex) and a mobile phase (20 mM ammonium acetate pH 6.9) running at 1 mL/min. Under these conditions, the retention times (R_T_) for NA and NAM were 7 and 19.9 min, respectively. The standard reaction medium consisted of 1 mM NAM and 0.14 μM of purified PolyNic in 100 mM phosphate buffer at pH 7.3. Reactions were stopped by the addition of trifluoroacetic acid to 1% (v/v). One unit of activity was defined as the amount of enzyme required to cleave 1 μmol of NAM releasing 1 μmol of NA in 1 min. The activity toward other NAM analogs was also determined by HPLC. Finally, the inhibition constants toward nicotinaldehyde and 5-bromo-nicotinaldehyde were obtained as previously described (Sanchez-Carron et al., 2013).

## Results

### Screening Assay Development

In order to establish a simple whole-cell method for nicotinamidases, the first step was to test the two previously described methods for detecting pyrazinamidase activity with POA and AFS. In the first method, used for detecting PZA activity in *Enterobacteriaceae*, a wireloop of an overnight bacterial culture on Tryptic Soy Broth is suspended in 0.2 mL of a 0.5% PZA solution, incubated for 18 h at 37°C, and finished by the addition of two drops of a 1% freshly prepared AFS solution to the culture (Pignato and Giammanco, 1992). In the second method, *E. coli* cells are cultured overnight in LB containing 8 mM PZA, centrifuged for 3 min at 13800 *g*, washed twice with 150 mM NaCl in 100 mM Tris-HCl pH 7.5, re-suspended in 8 mM with PZA, incubated at 37°C for 120 min, centrifuged again, revealed with 0.05 volumes of 500 mM AFS and, finally, the change in OD_480_ compared with a standard curve (Frothingham et al., 1996). However, when both methods were tested with a previously cloned *O. iheyensis* nicotinamidase (OiNic) in *E. coli* Rosetta 2 (Sanchez-Carron et al., 2013), several drawbacks came out. In the first method, the interferences produced with the culture medium gave faint and not very reliable colors, whereas in the second, the need of several steps, centrifugations and, especially the precipitation of POA-ferrous ion complex at pH > 6 hinders the correct spectrophotometric quantification. Thus, a more simple method suitable for HTS was developed by just resuspending the LB overnight OiNic *E. coli* pellets in a solution with 20 mM PZA and 1% AFS dissolved in MilliQ^®^ water at 37°C. The red color corresponding to POA/AFS complex was developed faster than in the above-described methods, just in 1 h (Supplementary Figure S1A), being this color even more intense after an overnight incubation, without any discernible color in control wells (Supplementary Figure S1B). Thus, to minimize the time of the assay for HTS, a clone that gave red color after 1 h was considered as a pyrazinamidase positive clone.

To test the sensitivity of the assay, the method was tested with different OiNic mutants (Sanchez-Carron et al., 2013). Clear differences between wild-type (OiNic Wt), K104A (total loss of activity), and E65H (less activity than the wild-type) mutants were observed, giving rise to a gradient of colors, depending on how such mutation affects the activity (**Figure 1C**). Nevertheless, when NAM is used for this screening instead of PZA, it is difficult to discriminate between more or less active clones, since a faint orange color is formed inside cells and not in the reaction medium (data not shown), making impossible to discriminate between them and control cells, even after centrifugation and subsequent detection in a microplate spectrophotometer.

**FIGURE 2:**
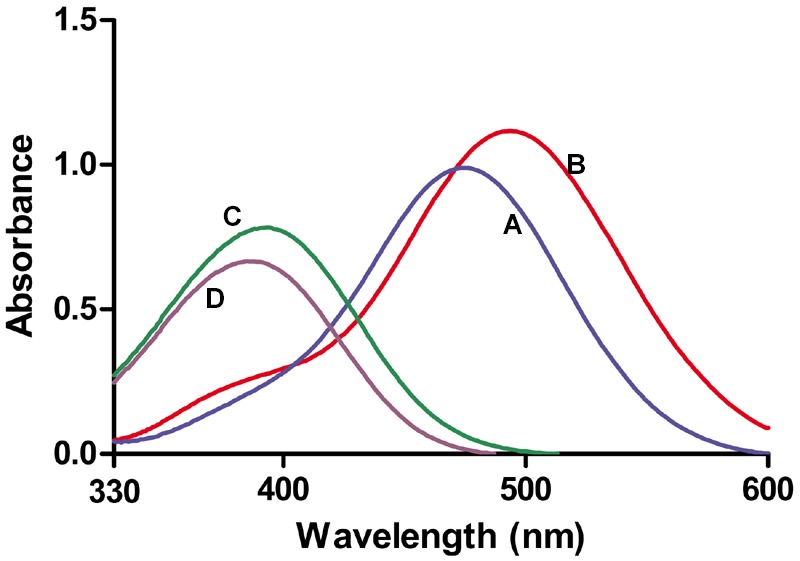
**Absorbance spectra obtained with nicotinamidase substrates and products in presence of sodium nitroprusside (SNP). (A)** Pyrazinoic acid-nitroprusside complex. **(B)** PZA-nitroprusside complex. **(C)** NAM-nitroprusside complex. **(D)** Nicotinic acid-nitroprusside complex. All nicotinamidase substrates and products were at 0.25 mM and SNP at 0.75% (w/v).

**FIGURE 3:**
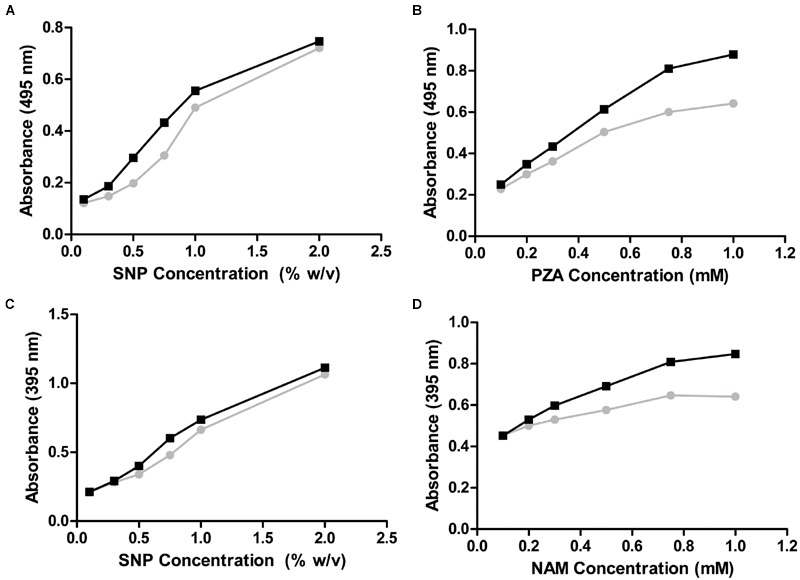
**Optimization of the whole-cell SNP method. (A,B)** PZA as substrate. **(A)** PZA was kept constant at 0.3 mM while SNP concentration was changed. **(B)** PZA concentration was modified at 0.75% SNP. **(C,D)** NAM as substrate. **(C)** NAM was kept constant at 0.3 mM while SNP concentration was adjusted. **(D)** NAM concentration was modified at 0.75% SNP. OiNic Wt clones are shown in black and control clones in gray.

Thus, only nicotinamidases with activity toward PZA could be screened with the AFS method. To circumvent this limitation, another activity assay was developed to be also used with NAM, using a different stable iron salt, SNP. This salt at neutral-basic pHs not only reacted with POA (**Figure 2**, curve A; 𝜀_475nm_ = 4374 M^-1^ cm^-1^) but also with PZA (**Figure 2**, curve B; 𝜀_495nm_ = 4549 M^-1^ cm^-1^). Surprisingly, it also gave appreciable color with both NAM (**Figure 2**, curve C; 𝜀_395nm_ = 3125 M^-1^ cm^-1^) and its corresponding acid (NA) (**Figure 2**, curve D; 𝜀_385nm_ = 2688 M^-1^ cm^-1^). To explore these different reactions (colors) of nicotinamidase substrates/products with SNP, which have never been used for whole-cell screening of this enzyme, different optimization conditions were tested with NAM and PZA as substrates. Since the change in color in the PZA/SNP method from red to orange was more evident, we started to optimize conditions with it. In order to see the decrease in the color of the wells with the naked eye, lower concentrations of PZA were needed (**Figure 3A**), when compared with the PZA/AFS method. At 0.3 mM PZA, the difference in color between OiNic Wt (**Figure 3A**, squares) and control clones (**Figure 3A**, circles) was more evident at 0.75% SNP. Once the SNP concentration was adjusted, different concentrations of the substrate were tested at 0.75% SNP in order to get the highest resolution. The difference in absorbance at 495 nm increased with PZA concentration up to 1 mM (about 0.2 absorbance units), tending toward a plateau (**Figure 3B**). On the other hand, the differences in the case of NAM were less evident by the naked eye due to the change in color from yellow to pale yellow, but still spectrophotometrically measurable. Again, 0.75% SNP (**Figure 3C**) and 1 mM NAM (**Figure 3D**) gave the best color contrast at 395 nm (also about 0.2 absorbance units). However, whereas substrate concentrations were decreased down to 1 mM in the SNP method, the revealing time increased up to 4 h to see measurable differences in color.

Collectively, these results show that this new SNP assay could be used as a re-screening method in order to find not only highly active pyrazinamidases, but also to easily distinguish among more or less active nicotinamidases in a quantitative (spectrophotometric) way.

### Screening of Metagenomic Libraries

**FIGURE 4:**
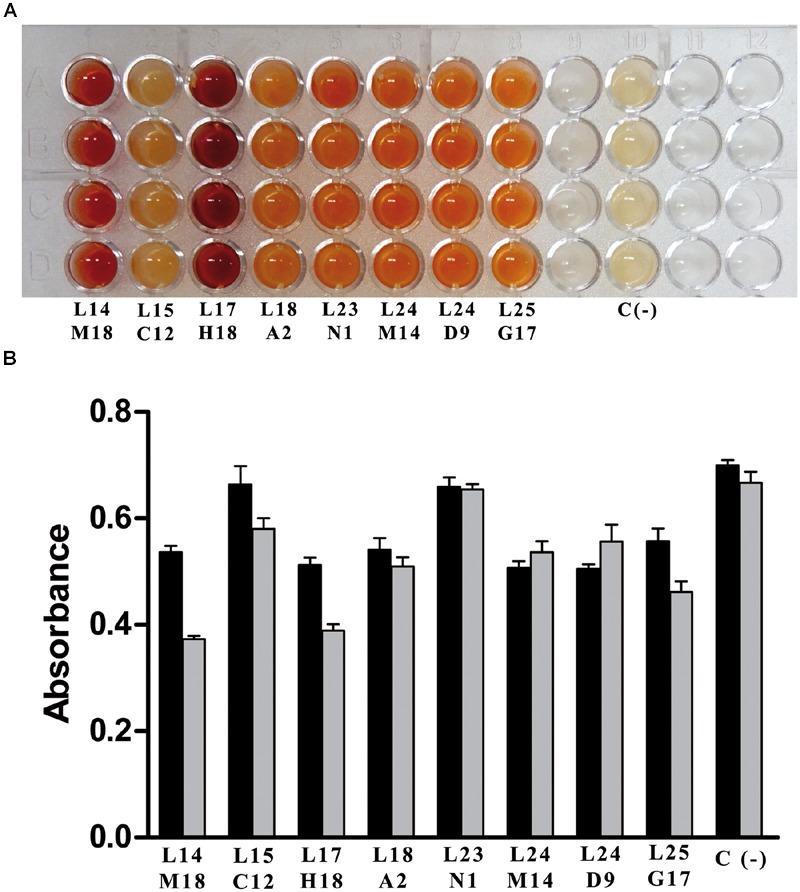
**Screening of mesophilic marine bacteria (MB) polygenomic library. (A)** Positive fosmid clones found in MB polygenomic library with the PZA/AFS method. **(B)** Positive fosmid clones found in MB polygenomic library rescreened with the SNP method with NAM (black bars) and PZA (gray bars) as substrates. Legends below bars reflect the position of the positive clones in their corresponding master plates. Negative controls (C-) are empty control cells (*E. coli* EPI300-T1 cells).

Once the two new whole-cell screening methods were optimized with a known nicotinamidase (OiNic) (Sanchez-Carron et al., 2013), a fosmid library obtained from mesophilic MB polygenomic library was first screened with the PZA/AFS method. After induction with the autoinduction solution (Epicentre, USA) (Supplementary Figure S2), only 8 over 8832 fosmid clones screened were positive with a different degree of activity, as shown by their different color intensity (**Figure 4A**). These clones were then re-screened with NAM (**Figure 4B**, black bars) and PZA (**Figure 4B**, gray bars) with the SNP method, confirming not only the presence of pyrazinamidase activity but also its correlation with the nicotinamidase activity.

**FIGURE 5:**
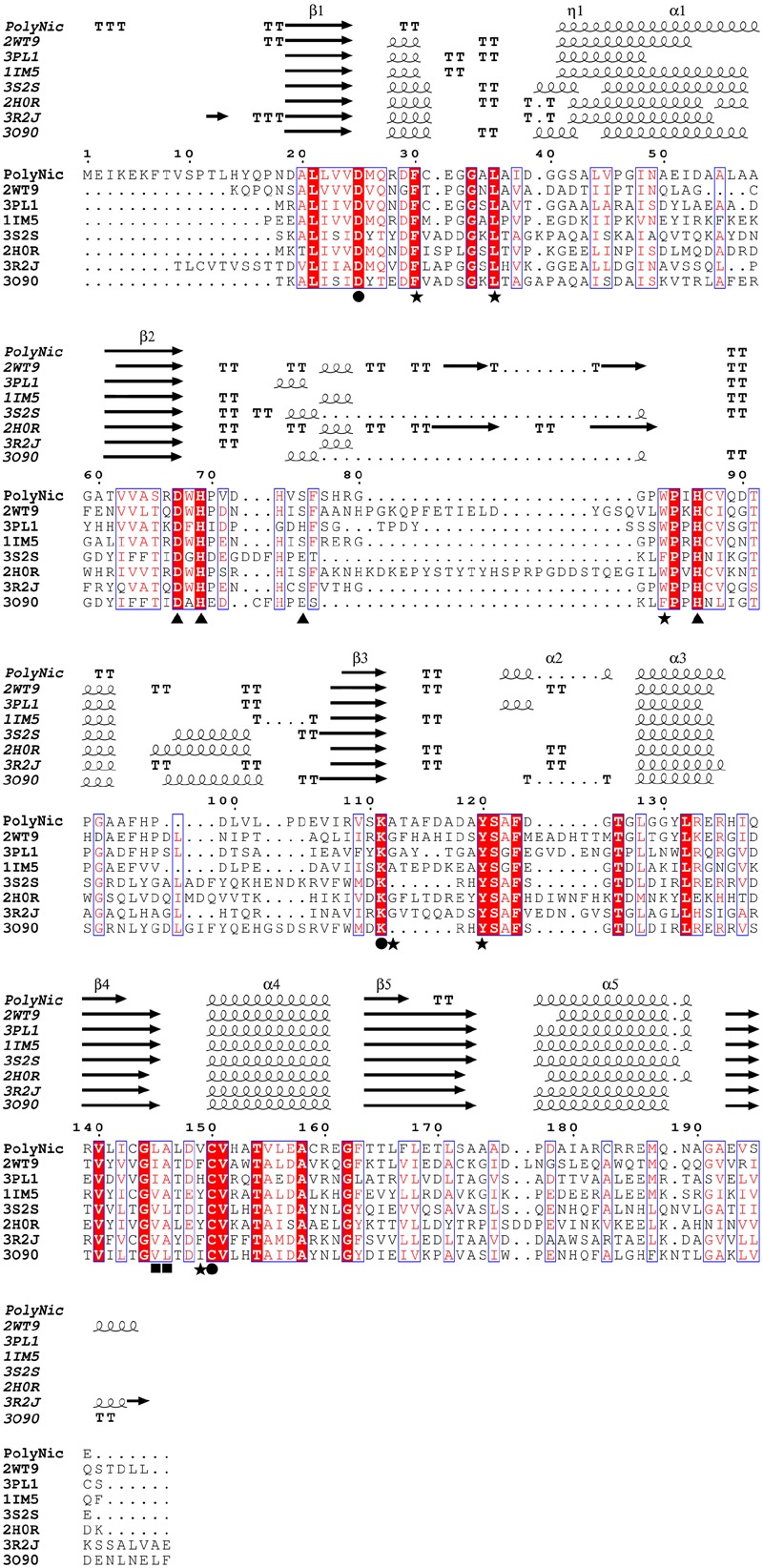
**Multiple sequence alignment between PolyNic and related crystallized nicotinamidases.** Strictly conserved residues have a red background and similar residues are marked with a blue square. Springs and arrows represent helices and strands of the secondary structure, respectively. Strict β-turns are represented as TT. Important residues for catalysis (●), *cis*-peptide bond (■), metal ion binding (▲) and the hydrophobic cavity where NAM and metal ion bind are also indicated (★). Four letter codes in the name of the sequences are PDB codes.

Clear differences between clones with respect to both activities were observed (**Figure 4B**), and for a proof-of-principle, one of the clones with similar high nicotinamidase and pyrazinamidase activity (**Figure 4B**; L24 M14) was selected to be sequenced, since it showed the most balanced decrease in absorbance with both substrates compared to the control. BLAST analysis of the amino acid sequence obtained from this polygenomic nicotinamidase (PolyNic) (**Figure 5**) showed a 91% identity with a nicotinamidase found in *Halomonas* sp. HL-48 (UniProt code: A0A0P7YXE9). In addition, PolyNic also showed variable similarity (25–46%, Supplementary Table S1) with the crystallized nicotinamidases found in the bibliography (**Figure 5**) (Du et al., 2001; Hu et al., 2007; Fyfe et al., 2009; French et al., 2010a; Gazanion et al., 2011; Liu et al., 2011; Petrella et al., 2011).

The alignment also revealed that PolyNic contained all the conserved catalytic triad residues of the cysteine-hydrolases family (**Figure 5**, filled circles): a catalytic cysteine at position 150 (C150, PolyNic numbering), an aspartate at position 25 (D25) and a lysine at position 111 (K111) (French et al., 2010b; Sanchez-Carron et al., 2013; Ion et al., 2014; Sheng and Liu, 2014). Furthermore, PolyNic showed the characteristic *cis*-peptide bond (**Figure 5**, positions L145 and A146, filled squares), which is invariably preceded by a conserved glycine (G144), and the conserved specific metal ion binding, which includes one aspartate (D67) and two histidines (H69 and H86) (**Figure 5**, filled triangles). Depending on the metal ion and on the structural conformation of the protein, a fourth residue may be implicated in the metal ion binding (Sanchez-Carron et al., 2013). This residue can be a glutamic acid, a histidine or a serine, being the last one the case of PolyNic (S75). Other residues also involved in the formation of the hydrophobic cavity, where NAM and the ion metal bind (Zhang et al., 2008; Sanchez-Carron et al., 2013) are also present in PolyNic, such as F30, L36, W83, A112, Y120, and V149 (**Figure 5**, filled stars; Supplementary Table S2).

### Substrate Specificity and Kinetic Parameters

PolyNic functional characterization was carried out after cloning its sequence into pET46 Ek/LIC vector. The recombinant clone with the highest expression was induced with 0.5 mM IPTG for 16 h at 25°C in TB medium with constant agitation and purified to homogeneity from *Escherichia coli* Rosetta 2 DE3 cells by a simple procedure based in a Ni^2+^-chelating affinity chromatography and a gel filtration step onto a Superdex 200, as described in the “Materials and Methods” Section. The molecular mass of the purified protein was determined by SDS-PAGE (∼23 kDa), HPLC/ESI/ion trap (24.1 kDa) and gel filtration in a Superdex 200 10/300 GL column (50.3 kDa). These experiments confirmed its dimeric nature.

**FIGURE 6:**
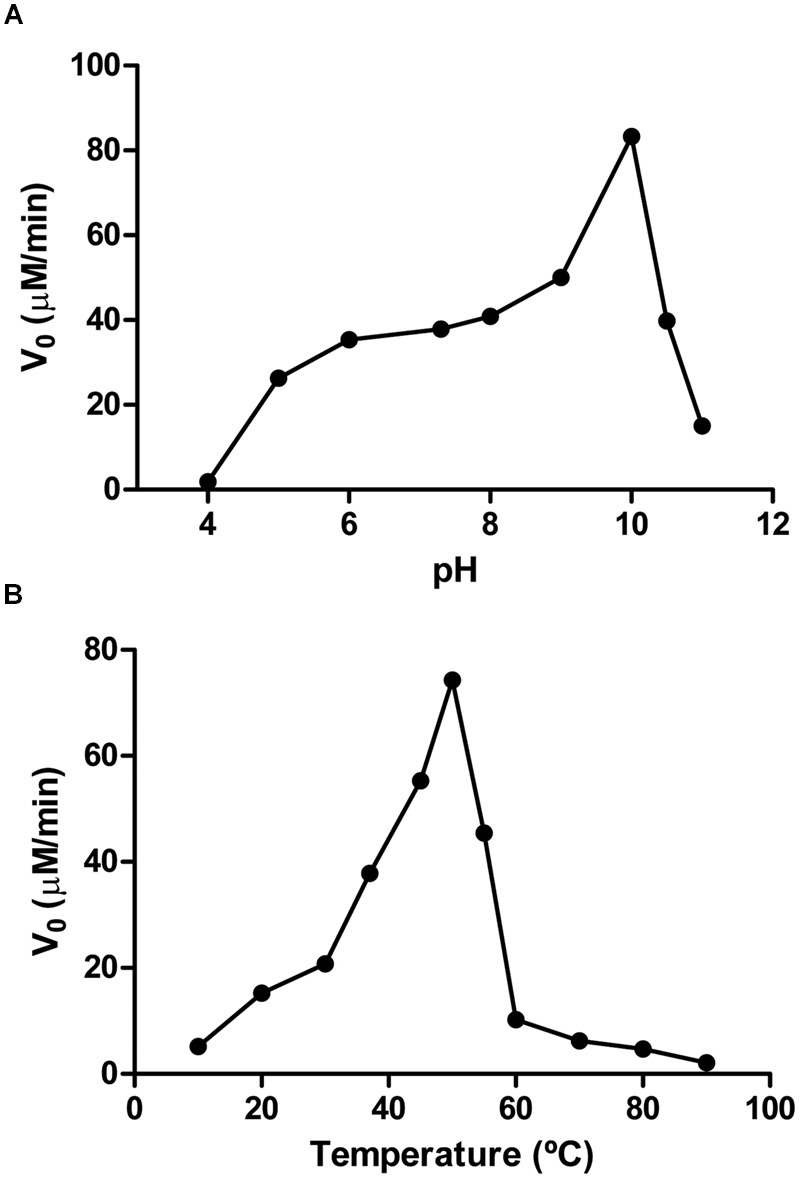
**Effect of pH and temperature on PolyNic activity. (A)** pH profile. Assay conditions were 1 mM NAM and 0.14 μM PolyNic in different 100 mM buffers at 37°C. Buffers used were sodium acetate pH 4.0–5.0, sodium phosphate pH 6.0–7.3, Tris-HCl pH 8.0, glycine pH 9.0–10.0, CAPS pH 10.5–11.0. **(B)** Temperature profile. Assay conditions were the same as above but at pH 7.3 and different temperatures (10–90°C).

The enzyme activity was both pH- and temperature-dependent. The activity increased as pH increases from pH 5.0 to pH 10.0 (**Figure 6A**), being the first nicotinamidase described with its optimal activity at that basic pH, since the highest optimum pH described in the bibliography was pH 8.0 for the *Lactobacillus arabinosus* 17-5 nicotinamidase (Hughes and Williamson, 1953). PolyNic exhibited its maximum activity at a temperature of 50°C (**Figure 6B**), which is 5 to 20°C higher than those described for other nicotinamidases (Pardee et al., 1971; Yan and Sloan, 1987; Zhang et al., 2008; Sanchez-Carron et al., 2013). However, and for comparison purposes, kinetic parameters were carried out at pH 7.3 and 37°C, as generally accepted in the bibliography (Zhang et al., 2008; French et al., 2010b; Sanchez-Carron et al., 2013).

The activity of PolyNic was studied toward NAM and some of its derivatives (**Table 1**), which include PZA, 5-methylnicotinamide and two nicotinate esters (methylnicotinate and ethylnicotinate). PolyNic showed a quite similar catalytic efficiency for PZA (*k*_cat_/*K*_M_ 69.9 mM^-1^s^-1^) and for NAM (*k*_cat_/*K*_M_ 102.0 mM^-1^ s^-1^). Thus, PolyNic is the first described bacterial nicotinamidase with these characteristics, since the rest of characterized nicotinamidases have a marked preference for NAM instead of PZA (more difference between their catalytic efficiencies), including that of *Mycobacterium tuberculosis* (Zhang et al., 2008; French et al., 2010b; Sanchez-Carron et al., 2013). Furthermore, PolyNic also showed activity toward 5-methylnicotinamide, although with a lower catalytic efficiency (*k*_cat_/*K*_M_ 18.9 mM^-1^ s^-1^) compared to NAM and PZA, but it was still 2.5-fold higher than that described before for OiNic (*k*_cat_/*K*_M_ 7.5 mM^-1^ s^-1^) (Sanchez-Carron et al., 2013). Interestingly, the enzyme was active toward methylnicotinate (*k*_cat_/*K*_M_ 19.7 mM^-1^ s^-1^), with the highest ever-described catalytic efficiency. In fact, PolyNic is 293-fold more active toward methylnicotinate than OiNic (Sanchez-Carron et al., 2013). Finally, ethylnicotinate was a very poor substrate for PolyNic (*k*_cat_/*K*_M_ 1.0 mM^-1^s^-1^) as usual in this kind of enzymes, but still ninefold higher than for OiNic (Sanchez-Carron et al., 2013).

**Table 1:** Kinetic parameters of PolyNic toward different substrates.

Substrate	*K*_m_, μM	*k*_cat_, s^-1^	*k*_cat_/*K*_m_, mM^-1^ s^-1^
Nicotinamide (NAM)	49 ± 2	5.0 ± 0.1	102.0
Pyrazinamide (PZA)	103 ± 10	7.2 ± 0.2	69.9
5-methyl-NAM	435 ± 15	8.2 ± 0.5	18.9
Methylnicotinate	355 ± 30	7.0 ± 0.4	19.7
Ethylnicotinate	1092 ± 105	1.1 ± 0.1	1.0

### Applicability of the Whole-Cell High-Throughput Screening for Inhibitor Testing

**FIGURE 7:**
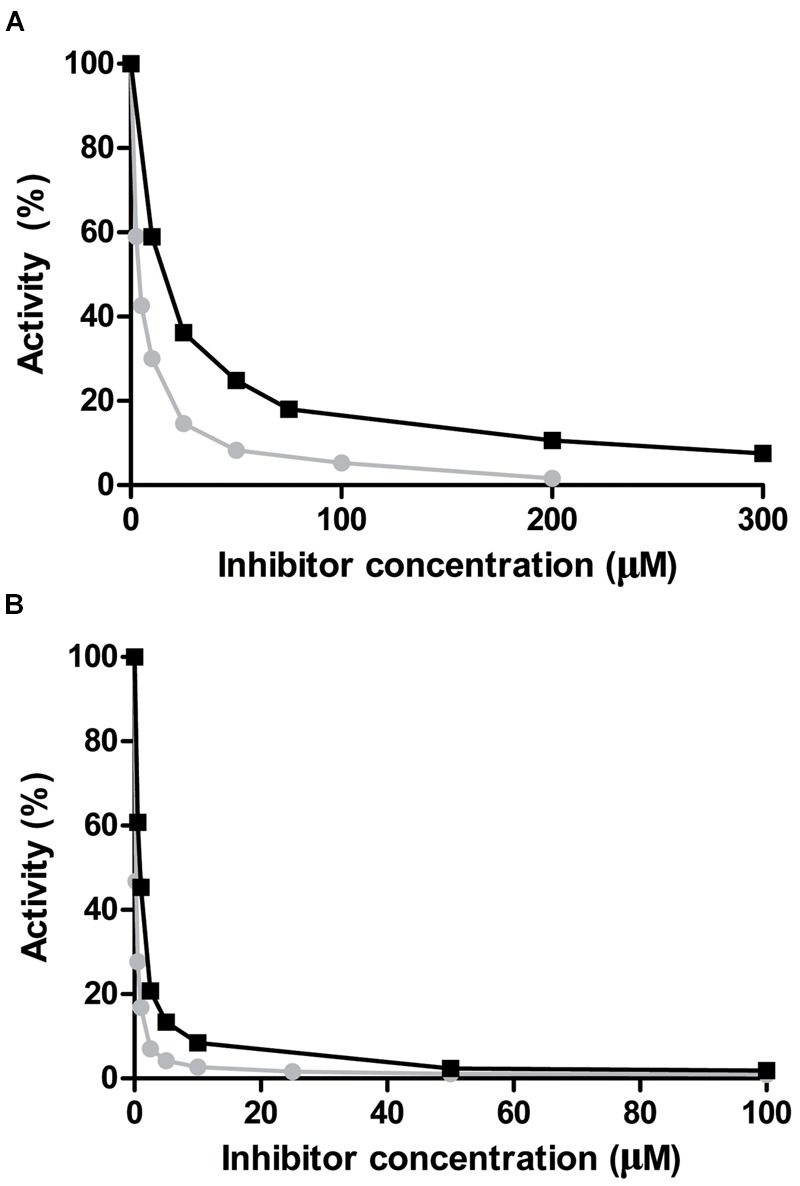
**Inhibition of PolyNic by nicotinaldehydes. (A)** Inhibition of PolyNic by nicotinaldehyde (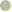) and 5-bromo-nicotinaldehyde (■) using NAM as substrate. **(B)** The same as **(A)** but with PZA as substrate. Reactions were carried out at 37°C, 1 mM substrate (NAM or PZA) and 0.14 μM PolyNic in 100 mM phosphate buffer pH 7.3. Morrison’s equation was used to fit data and to obtain the *K*_i_ values, as previously described (Sanchez-Carron et al., 2013).

Nicotinaldehydes have been reported to act as good competitive inhibitors for several nicotinamidases (French et al., 2010b; Sanchez-Carron et al., 2013). Thus, recombinant PolyNic was tested with nicotinaldehyde and 5-bromo-nicotinaldehyde, toward both substrates, NAM (**Figure 7A**) and PZA (**Figure 7B**), respectively. The *K*_i_ value for nicotinaldehyde with NAM was found to be 0.18 μM (**Figure 7A**; circles), a similar value to that found in *Borrelia burgdorferi* nicotinamidase (0.11 μM) (French et al., 2010b). The *K*_i_ value for 5-bromo-nicotinaldehyde with NAM was higher (0.72 μM) (**Figure 7A**; squares) than that of nicotinaldehyde, and similar to that found for the *Plasmodium falciparum* nicotinamidase (0.57 μM) (French et al., 2010b). Interestingly, the *K*_i_ values for nicotinaldehyde and 5-bromo-nicotinaldehyde obtained with PZA were 0.015 and 0.07 μM, respectively (**Figure 7B**), which are much lower than those obtained using NAM as substrate. These values could not be compared since no data on nicotinaldehyde inhibition with PZA have been published.

With the *K*_i_ values described above, our goal was to develop the first whole-cell screening method for testing nicotinamidase inhibitors using the PZA/AFS and NAM/SNP methods. Thus, different concentrations of nicotinaldehyde and 5-bromo-nicotinaldehyde (0–1500 μM) were added to each well inoculated with PolyNic in *E. coli* Rosetta 2 before adding the revealing solution (20 mM PZA/1% AFS or 1 mM NAM/0.75% SNP). Surprisingly, SNP was not suitable for inhibitor screening because the difference of absorbance between the untreated and the treated wells was not enough to rely on this method with both substrates. Furthermore, at the highest concentration of the inhibitors, the absorbance was greater than expected in comparison with the other wells, probably because of a reaction between SNP and the inhibitors themselves (data not shown).

**FIGURE 8:**
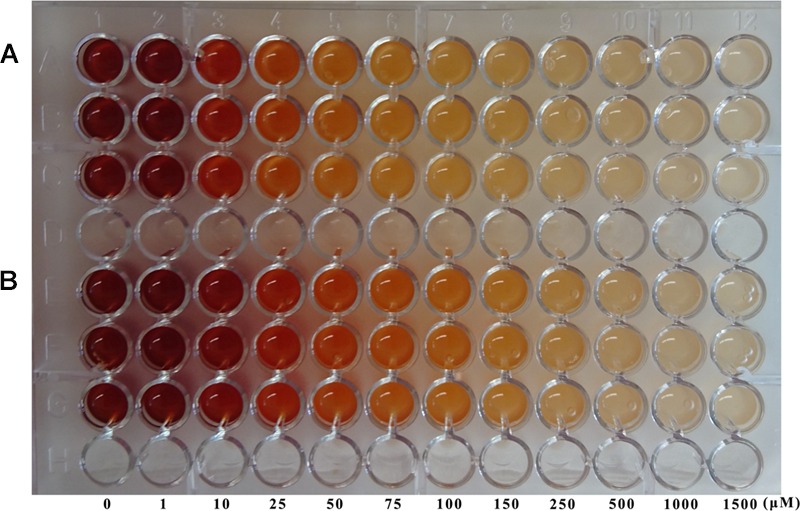
**Inhibitor testing using the new whole-cell screening method.** Nicotinamidase aldehyde inhibitors, nicotinaldehyde **(A)** and 5-bromo-nicotinaldehyde **(B)** were assayed with the PZA/AFS method. *E. coli* Rosetta 2 cells containing pET46b-PolyNic vector were incubated in presence of 20 mM PZA and 1% AFS for 1 h at 37°C with different inhibition concentrations.

However, in the case of the AFS method, it was possible to find a gradient of colors (from red, orange to uncolored) as the inhibitor concentration rose (**Figure 8**), finding by the naked eye that nicotinaldehyde (**Figure 8A**) is a better inhibitor than 5-bromo-nicotinaldehyde (**Figure 8B**), since color formation stops at lower concentrations with nicotinaldehyde (75–100 μM) than with 5-bromo-nicotinaldehyde (250–500 μM) (**Figure 8**), which correlates well with the above-described kinetic results. These results make this whole-cell PZA/AFS assay a simple HTS qualitative prescreening method for nicotinamidase inhibitors.

## Discussion

Nicotinamidase activity is usually followed by reverse phase HPLC using its natural substrate (NAM), or continuously monitored following one of the two products of the nicotinamidase reaction (ammonia) in a coupled assay with bovine or recombinant glutamate dehydrogenases (GDH) (French et al., 2010b; Sanchez-Carron et al., 2013). These latter enzymes catalyze the reaction of α-ketoglutarate, ammonia, and NAD(P)H to form glutamate and the oxidized dinucleotide with the concomitant decrease in absorbance (𝜀_340_ = 6200 M^-1^ cm^-1^). While the latter method could be adapted for HT screening, it is not suitable for screening large metagenomic or directed evolution libraries, due to the coupled enzyme costs. To solve the above-mentioned problems, a new whole-cell method is proposed based on the substrate promiscuity of nicotinamidases. In fact, these enzymes are also able to use NAM derivatives, including PZA (**Figure 1B**). This latter compound was first commercialized with the name of Aldinamide by Lederle Laboratories (American Cyanamid, Wayne, NY, USA) for the treatment of *Mycobacterium tuberculosis* infections. In combination with isoniazid (INH) and rifampin, PZA allowed the conventional 9-month tuberculosis treatment regimen to be shortened to 4–6 months (Aldrich et al., 2010). This pro-drug is converted into POA by *Mycobacterium tuberculosis* nicotinamidase (Kalinda and Aldrich, 2012). In order to determine blood and urine levels of the drug, a simple colorimetric method was developed, in which an orange–red complex with a maximum absorbance at 480 nm is formed in the presence of AFS (also known as Mohr’s salt) after PZA in the blood extract was hydrolyzed with alkali to the corresponding salt of POA (Allen et al., 1953). This reaction was later applied to determine PZA deamidase activity in animal tissues and to perform PZA susceptibility tests in *Mycobacterium tuberculosis* strains at acid pH, even when such microorganism does not grow well at pH 5.5, giving rise, in some cases, to inconsistent results between different labs (Wayne, 1974; Martin et al., 2006; Chang et al., 2011). To reduce these contradictory results, a method was developed using *in vitro* synthesized pyrazinamidases (PZases) obtained after PCR amplification of *pnc*A gene of different *Mycobacterium* strains with their concomitant expression in a wheat germ system, followed by colorimetric detection of the corresponding POA generated with AFS (Zhou et al., 2011; Li et al., 2014). However, the latter multi-step method is not suitable for HTS. In addition, the two previously reported whole-cell methods described for detecting nicotinamidase activity (Pignato and Giammanco, 1992; Frothingham et al., 1996), when tested with a previously cloned *O. iheyensis* nicotinamidase (OiNic) in *E. coli* Rosetta 2 (Sanchez-Carron et al., 2013), showed several drawbacks. In the first method (Pignato and Giammanco, 1992), the interferences produced with the culture medium gave faint and not very reliable colors, whereas in the second (Frothingham et al., 1996), the need of several steps, centrifugations and, especially the precipitation of POA-ferrous ion complex at pH > 6 hinders the correct spectrophotometric quantification. Thus, a more simple and fast (just in 1 h, Supplementary Figure S1A) method suitable for HTS was developed by just resuspending the LB overnight OiNic *E. coli* pellets in a solution with 20 mM PZA and 1% AFS dissolved in MilliQ^®^ water at 37°C. The intensity of the color in the wells was also related to the activity expressed by the cells. A gradient of colors was observed between wild-type (OiNic Wt), K104A (total loss of activity), and E65H (less activity than the wild-type) mutants (**Figure 1C**). However, such differences in color were unreliable when NAM was used as substrate for this screening instead of PZA, making impossible to discriminate between mutants and control cells, even after centrifugation, and subsequent detection in a microplate spectrophotometer.

This limitation for screening enzymes highly active toward NAM as substrate was solved using SNP, which reacts with both substrates (NAM and PZA) and also with both products (NA and POA) (**Figure 2**), but giving complexes with different absorption coefficients. These differences in color could be easily followed spectrophotometrically, giving rise to a new method that is able to quantify both nicotinamidase and pyrazinamidase activities of a selected clone.

These two new whole-cell screening methods were tested sequentially with a polygenomic library (8832 fosmid clones) for a proof-of-principle. Among the 8 positive clones obtained (**Figure 4A**), the one with the highest activity toward both substrates was selected (**Figure 4B**; L24 M14) to be sequenced, showing all the conserved amino acids of an authentic nicotinamidase (**Figure 5**). When this enzyme, named as PolyNic, was kinetically characterized, it showed the lowest ratio described in bibliography between catalytic efficiency for NAM and for PZA (1.5). This ratio is far from those described for the nicotinamidases of *Mycobacterium tuberculosis* (17.5) (French et al., 2010b), *Oceanobacillus iheyensis* (13.5) (Sanchez-Carron et al., 2013), and *Saccharomyces cerevisiae* (4.4) (French et al., 2010b). These balanced activities convert PolyNic into a flexible biocatalyst for use in analytical kits for the determination of sirtuin activity and in tests for finding new pro-drug NAM derivatives to treat infections caused by the pathogenic organisms described in the “Introduction” section.

In addition, when PolyNic was modeled with SwissModel using *Pyrococcus horikoshii* nicotinamidase coordinates as template (PhNic, PDB 1IM5) and structurally aligned with another two crystallized nicotinamidases with a serine as the fourth amino acid involved in metal-binding (e.g., *Acinetobacter baumannii* (AbNic, 2WT9) and *Pyrococcus horikoshii* (PhNic, 1IM5) nicotinamidases), the only difference found in the active center was the presence of a valine residue (V149 in PolyNic) rather than a phenylalanine (F158 in AbNic) or a tyrosine (Y132 in PhNic) (**Figure 5**, filled stars; Supplementary Figure S3; Supplementary Table S2). The absence of an aromatic ring at this position of the active center might be responsible for the balanced catalytic efficiency. However, more data are needed to test this hypothesis, since no catalytic efficiencies for both substrates have been described for AbNic and PhNic so far (Du et al., 2001; Fyfe et al., 2009). In fact, only AbNic catalytic efficiency for NAM is known (0.48 mM^-1^ s^-1^), having been found to be 212-fold lower than that of PolyNic. The latter enzyme (PolyNic) has also higher catalytic efficiency than the *Saccharomyces cerevisiae* PNC1 nicotinamidase (French et al., 2010b), which is the most used nicotinamidase in enzyme-coupled assays for identifying new sirtuin modulators (Hubbard and Sinclair, 2013) as possible pharmaceuticals in lifespan, cancer, obesity and neurodegenerative diseases. Thus, PolyNic could be a clear biotechnological alternative for a commercial nicotinamidase due to its high activity and expression, together with its simple and efficient purification procedure.

The kinetic characterization of PolyNic has also allowed us to develop the first high-throughput whole-cell method for prescreening of new nicotinamidase inhibitors by the naked eye. In this case, only the qualitative PZA/AFS method was reliable, finding that nicotinaldehyde (**Figure 8A**) is a better inhibitor than 5-bromo-nicotinaldehyde (**Figure 8B**), since color formation stops at lower concentrations with nicotinaldehyde, and supporting the kinetic data obtained (**Figure 7**).

Finally, the screening methods described in the present paper could be further optimized by changing the salts used to form the complexes with the enzyme substrates/products. For this, different divalent ions, such as cobalt, nickel or copper could be used. However, the type of salt has to be taken into account since, for example, the above metal ions in their perchlorate forms give insoluble complexes with POA (Magri et al., 1980). On the other hand, SNP could be replaced with a similar stable ferrous salt, such as potassium ferrocyanide, although the conditions (concentrations, time, etc) have to be optimized in the same way as described in the present paper. Further research is needed to find new complexes with NAM and PZA with the ultimate aim of developing a cheap and reliable PZA susceptibility test for the different *Mycobacterium tuberculosis* strains.

Interestingly, the SNP method could also be used to assay other enzymatic activities besides nicotinamidases. In fact, this method could permit the detection of new inhibitors of two enzymes, which are currently targets in cancer therapy (Cerna et al., 2012; Duarte-Pereira et al., 2016) – the human NAMPT (EC 2.4.2.12) and the nicotinate phosphoribosyltransferase (NaPRT) (EC 6.3.4.21), which use NAM and NA as substrates to convert them into NMN and nicotinic acid mononucleotide (NaMN), respectively. These enzymatic reactions produce a β-*N*-glycosidic bond with the pyridine nitrogen of both NAM and NA in presence of phosphoribosyl pyrophosphate (PRPP) and ATP. The absence of this nitrogen donor in the pyridine ring of NMN and NaMN hinders the formation of complexes with SNP. Thus, in presence of an inhibitor (e.g., GMX1778 for NAMPT), the color of the reaction medium will remain yellow because of the reaction of NAM or NA with SNP, whereas in the absence of an inhibitor, the reaction medium will become transparent. Hence, the potency of the inhibitor tested could be easily measured spectrophotometrically following the decrease in the absorbance at 395 nm for NAM or 385 nm for NA.

## Conclusion

We have developed two whole-cell methods for the screening of metagenomic/polygenomic libraries to discover new enzymes with balanced pyrazinamidase/nicotinamidase activities, using for this purpose the chemical property of POA and NAs to form colored complexes with the iron stable salts. After screening more than 8000 clones in a fosmid polygenomic library, one positive clone was selected with high and balanced nicotinamidase and pyrazinamidase activities, which could be a good candidate for biotechnological applications. In addition, its inhibition pattern with different nicotinaldehydes has allowed us to develop a new pre-screening method that could save time and costs in the search of promising drugs to fight against bacterial and parasitic human diseases (Lyme disease, Malta fever, malaria and infantile visceral leishmaniasis). Finally, the method could be also applied to screen for other enzymes with biomedical relevance that use NA or NAM as a substrate.

## Author Contributions

RZ-P, AGG-S and AS-F: Developed the assay. RZ-P, AGG-S, MJ, and PG: Conducted the experiments. MJ, PG, and AS-F: Designed the experiments. All authors interpreted the data, wrote the manuscript, and have given approval to the final version of the manuscript.

## Conflict of Interest Statement

The authors declare that the research was conducted in the absence of any commercial or financial relationships that could be construed as a potential conflict of interest.

## Footnotes

1 https://swissmodel.expasy.org/
