# Supplementary material for: Combined Whole-Cell High-Throughput Functional Screening for Identification of New Nicotinamidases/Pyrazinamidases in Metagenomic/Polygenomic Libraries
Source: Front Microbiol. 2016 Nov 29;7:1915. doi: 10.3389/fmicb.2016.01915 (PMC5147024; doi:10.3389/fmicb.2016.01915)
Supplement: Supplementary file 1 [file Presentation_1.pdf]

Supplementary Material

Combined whole-cell high-throughput functional screening for identification of new nicotinamidases/pyrazinamidases in metagenomic/polygenomic libraries

Authors:
Rubén Zapata-Pérez[†], Antonio Ginés García-Saura[†], Mohamed Jebbar[‡], Peter N. Golyshin[⊥, Δ,*], Álvaro Sánchez-Ferrer[†,§,*]

Affiliations:
†Department of Biochemistry and Molecular Biology-A, Faculty of Biology, Regional Campus of International Excellence "Campus Mare Nostrum", University of Murcia, Campus Espinardo, E-30100 Murcia, Spain
‡Univ Brest, CNRS, Ifremer, UMR 6197-Laboratoire de Microbiologie des Environnements Extrêmes (LM2E), Institut Universitaire Européen de la Mer (IUEM), rue Dumont d'Urville, 29 280 Plouzané, France
⊥School of Biological Sciences, Bangor University LL57 2UW, Bangor, Gwynedd, UK
Δ Immanuel Kant Baltic Federal University, 236040 Kaliningrad, Russia
§Murcia Biomedical Research Institute (IMIB-Arrixaca), 30120 Murcia, Spain

*Co-corresponding authors. Email: p.golyshin@bangor.ac.uk (P.N.G.), alvaro@um.es (A.S-F.)

CONTENTS:

Figure S1. Absence of background color in the pyrazinamide-ammonium ferrous sulfate method after 1 and 24 hours of incubation.
Figure S2. Influence of the autoinduction solution in color development during screening with the pyrazinamidase-ammonium ferrous sulfate method.
Figure S3. Structural alignment of modeled PolyNic compared to other related nicotinamidases.
Table S1. Similarity between PolyNic and different crystallized nicotinamidases.
Table S2. Comparative study of the residues involved in nicotinamidase activity.
References

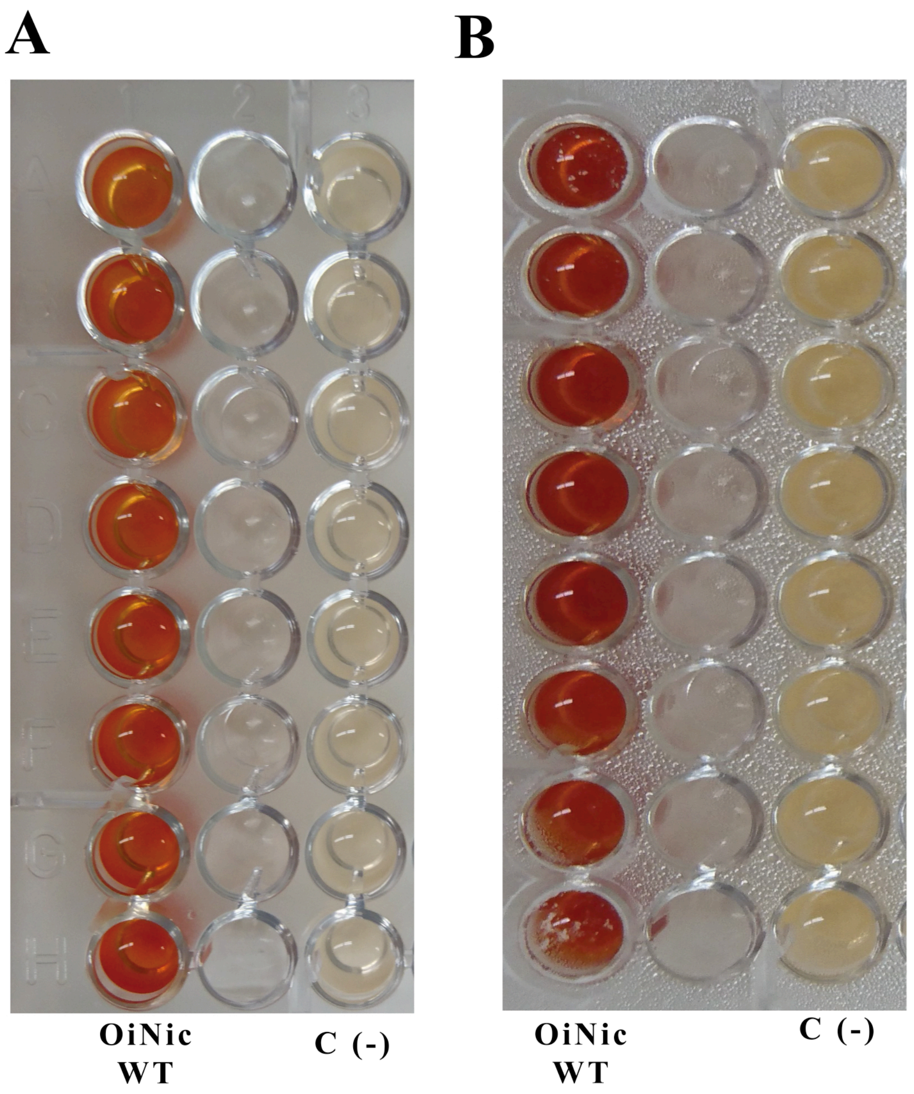

Figure S1 | Absence of background color in the pyrazinamide-ammonium ferrous sulfate method after 1 and 24 hours of incubation. (A) Orange-red color developed by a nicotinamidase positive clone (recombinant *Oceanobacillus iheyensis* nicotinamidase, OiNic) (Sanchez-Carron et al., 2013) after 1 hour. (B) Same as (A) but after 24 hours of incubation. Negative control cells (C-) were *E. coli* Rosetta 2 cells transformed with an empty pET28a vector.

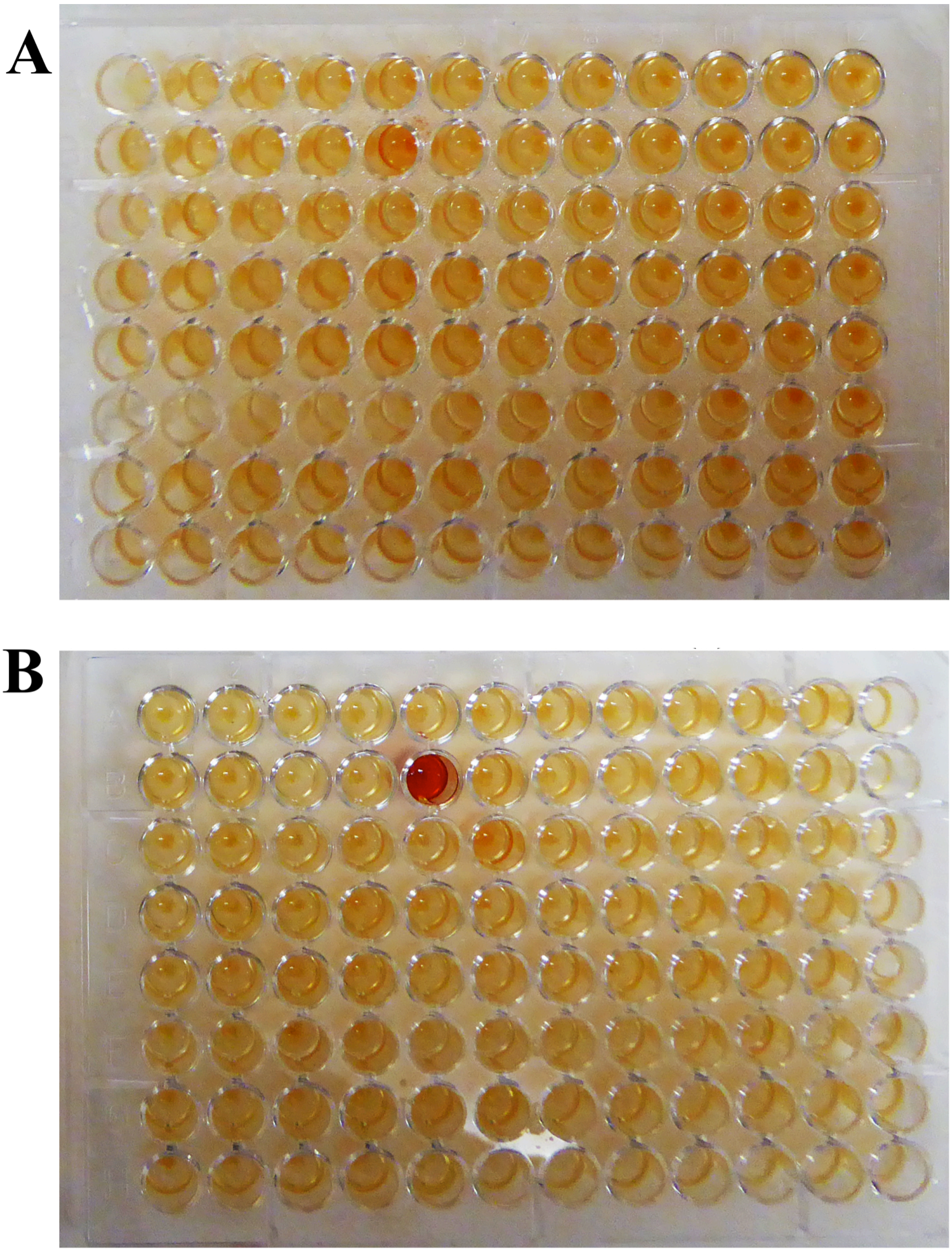

Figure S2 | Influence of the autoinduction solution in color development during screening with the pyrazinamide-ammonium ferrous sulfate method. (A) Plates were revealed after growing the fosmid clones in the absence (A) and in the presence (B) of the autoinduction solution (Epicentre, USA).

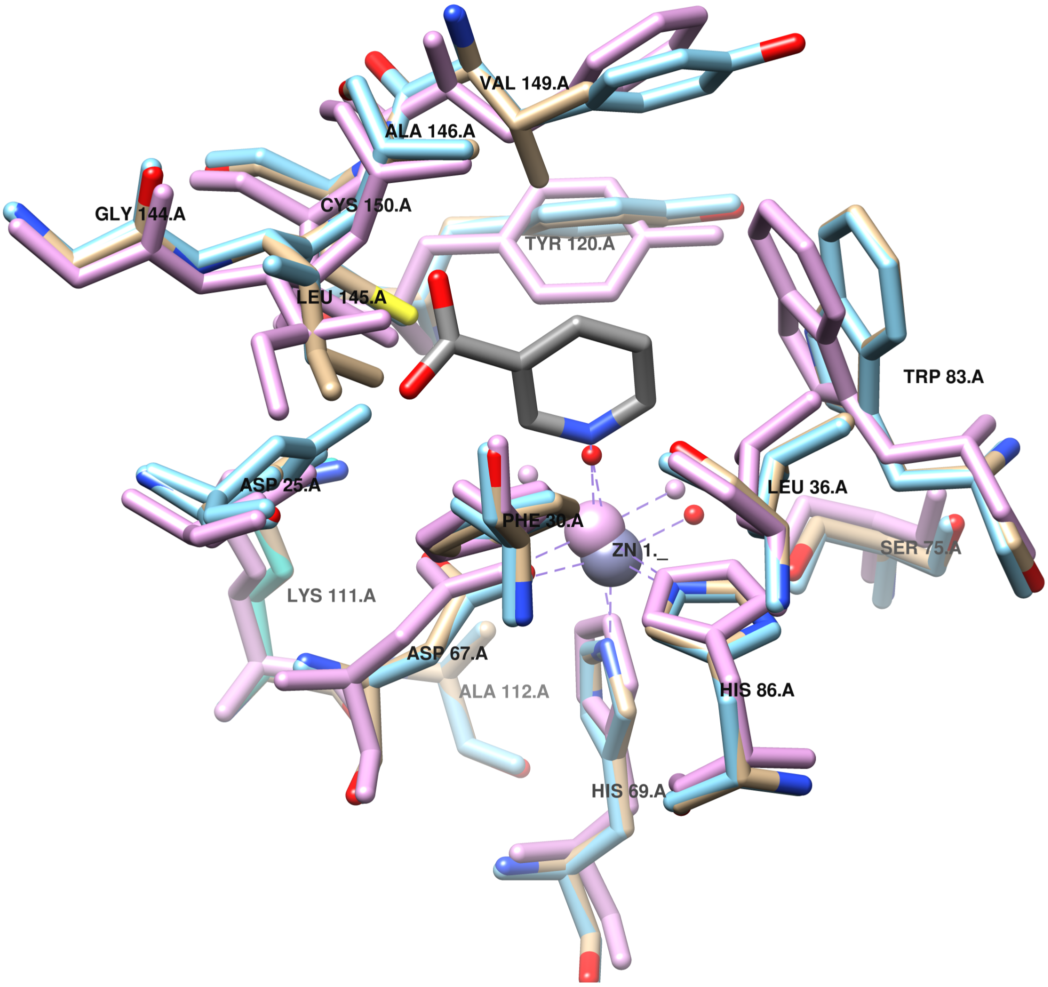

Figure S3 | Structural alignment of modeled PolyNic compared to other related nicotinamidases. PolyNic (tan) was modeled and structurally aligned with *Pyrococcus horikoshii* nicotinamidase *(Du et al., 2001)* (1IM5, cyan) and *Acinetobacter baumanii* nicotinamidase *(Fyfe et al., 2009)* (2WT9, magenta) as described in Materials and methods section. For simplicity, only labels of PolyNic are shown. The equivalent residues are described in Table S2. Nicotinic acid from 2WT9 is shown in gray.

Table S1 | Similarity between PolyNic and different crystallized nicotinamidases

Source microorganism	PDB code	Similarity	Reference
Pyrococcus horikoshii	1IM5	46%	*(Du et al., 2001)*
Leishmania infantum	3R2J	42%	*(Gazanion et al., 2011)*
Mycobacterium tuberculosis	3PL1	39%	*(Petrella et al., 2011)*
Acinetobacter baumanii	2WT9	36%	*(Fyfe et al., 2009)*
Saccharomyces cerevisiae	2H0R	30%	*(Hu et al., 2007)*
Streptococcus pneumonia	3O90	29%	*(French et al., 2010)*
Streptococcus mutans	3S2S	25%	*(Liu et al., 2011)*

Table S2. Comparative study of the residues involved in nicotinamidase activity

Residues	PolyNIC	PhNic* (1IM5)	AbNic (2WT9)
Catalytic triad	C150	C133	C159
	D25	D10	D16
	K111	K94	K114
Metal binding	D67	D52	D54
	H69	H54	H56
	H86	H71	H89
	S75	S60	S62
Cis-peptide bond oxyanion hole	G144	G127	G153
	L145	V128	I154
	A146	A129	A155
Active site forming residues	W83	W68	W86
	V149	Y132	F158
	Y120	Y103	Y123
	F30	F15	F21
	L36	L21	L27
	A112	A95	G115

*PhNic: *Pyrococcus horikoshii* nicotinamidase *(Du et al., 2001)*; AbNic: *Acinetobacter baumanii* nicotinamidase *(Fyfe et al., 2009)*

REFERENCES

Du, X., W. Wang, R. Kim, H. Yakota, H. Nguyen, and S.H. Kim. 2001. Crystal structure and mechanism of catalysis of a pyrazinamidase from Pyrococcus horikoshii. *Biochemistry*. 40:14166-14172. doi:10.1021/bi0115479

French, J.B., Y. Cen, A.A. Sauve, and S.E. Ealick. 2010. High-resolution crystal structures of Streptococcus pneumoniae nicotinamidase with trapped intermediates provide insights into the catalytic mechanism and inhibition by aldehydes. *Biochemistry*. 49:8803-8812. doi:10.1021/bi1012436

Fyfe, P.K., V.A. Rao, A. Zemla, S. Cameron, and W.N. Hunter. 2009. Specificity and mechanism of Acinetobacter baumanii nicotinamidase: implications for activation of the front-line tuberculosis drug pyrazinamide. *Angew. Chem. Int. Ed. Engl.* 48:9176-9179. doi:10.1002/anie.200903407

Hu, G., A.B. Taylor, L. McAlister-Henn, and P.J. Hart. 2007. Crystal structure of the yeast nicotinamidase Pnc1p. *Arch. Biochem. Biophys.* 461:66-75. doi:10.1016/j.abb.2007.01.037

Liu, X., H. Zhang, X.J. Wang, L.F. Li, and X.D. Su. 2011. Get phases from arsenic anomalous scattering: de novo SAD phasing of two protein structures crystallized in cacodylate buffer. *PLoS One*. 6:e24227. doi:10.1371/journal.pone.0024227

Petrella, S., N. Gelus-Ziental, A. Maudry, C. Laurans, R. Boudjelloul, and W. Sougakoff. 2011. Crystal structure of the pyrazinamidase of Mycobacterium tuberculosis: insights into natural and acquired resistance to pyrazinamide. *PLoS One*. 6:e15785. doi:10.1371/journal.pone.0015785

Sanchez-Carron, G., M.I. Garcia-Garcia, R. Zapata-Perez, H. Takami, F. Garcia-Carmona, and A. Sanchez-Ferrer. 2013. Biochemical and mutational analysis of a novel nicotinamidase from Oceanobacillus iheyensis HTE831. *PLoS One*. 8:e56727. doi:10.1371/journal.pone.0056727
